# Immortalization of Human Keratinocytes Using the Catalytic Subunit of Telomerase

**DOI:** 10.1134/S1607672921010014

**Published:** 2021-03-10

**Authors:** A. K. Beilin, N. G. Gurskaya, N. A. Evtushenko, E. V. Alpeeva, A. V. Kosykh, V. V. Terskikh, A. V. Vasiliev, E. A. Vorotelyak

**Affiliations:** 1 Koltzov Institute of Developmental Biology, Russian Academy of Sciences, Moscow, Russia; 2 Pirogov Russian National Research Medical University, Moscow, Russia; 3 Moscow State University, Moscow, Russia

**Keywords:** human keratinocytes, telomerase, transduction, transformation, immortalization, cell culture, skin equivalent, immunodeficient mice, tumorigenicity

## Abstract

A new stable line of human keratinocytes was obtained. The cells have altered morphology, both abnormal chromosomal composition and expression of keratinocyte markers, do not show contact inhibition, could be cultured in various media and have limited stratification ability in vitro. Upon transplantation into nude mice the cells have tumorigenic properties.

The main controllers of cellular aging are telomeres—the regions at the ends of chromosomes that in primary somatic cells are gradually shortened with each division. In germ and some somatic stem cells, the ribonucleic complex, telomerase, is active, the catalytic subunit of which (hTERT) synthesizes telomeric repeats, and manifests its functions, being heterologically expressed in cells of different types [1]. However, ectopic expression of hTERT alone is insufficient for immortalization of keratinocytes, which require activation of cyclin-dependent kinases to pass checkpoints in the mitotic cycle [2, 3]. Due to this fact, only several lines of spontaneously immortalized keratinocytes are available to researchers: NM1, HaCaT, and NIKS [4, 5]. Earlier, it was shown that the expression of hTERT cDNA in primary keratinocytes can lead to the production of stable cell lines [4], apparently due to additional spontaneous transformation.

In this study, we transformed the primary human keratinocytes using hTERT, which allowed us to obtain a stable cell line. The purpose of the subsequent work was to study the properties of the obtained cells.

Isolation and cultivation of primary keratinocytes was described previously [6]. Immortalization was performed by transduction of a lentiviral vector carrying the hTERT cDNA. The selection of immortalized cells was performed using selection for puromycin resistance.

A cytogenetic analysis of cells was performed by differential G-banding.

Telomerase activity was assessed using the TRAPEZE  kit (S7710) according to the manufacturer’s recommendations.

Phase contrast microscopy of cell cultures was performed with an EVOS FL AUTO microscope.

Cells were transplanted into the testes of immunodeficient mice according to the standard protocol in an SPF vivarium [7].

The creation of skin equivalents and the preparation of cryosections were carried out as described previously [6]. In one of the groups, the CnT-Prime Airlift culture medium (Ztn-Bio, United States) was replaced with DMEM/F12 with 10% serum, which was supplemented with ITS (Insulin–Transferrin–Selenium additive) and EGF (recombinant human epidermal growth factor) (Gibco, United Statets).

Immunofluorescent staining of cell cultures and cryosections was carried out according to the method described earlier [8].

Confocal imaging was performed using an LSM 880 laser scanning confocal microscope based on Axio Observer.Z1.

The culture of primary keratinocytes before the immortalization procedure had the “cobblestone pavement” morphology typical of keratinocytes (Fig. 1a). After lentiviral transduction and selection in an antibiotic-containing medium, a population of cells with altered morphology was detected in the culture. These cells were smaller in diameter, had a spherical shape, and formed a small number of large processes (Fig. 1b). After the formation of a monolayer, the cells showed no contact inhibition. After several passages, these cells completely displaced other keratinocytes, forming a morphologically homogeneous population that persisted for more than 20 passages. In the DMEM/F12 medium with serum, which contained a higher concentration of calcium ions than the CnT-07 medium, which was used at the first stages of the study, the morphology and growth characteristics changed. The cells became more flattened but, despite the continual growth, they did not form a monolayer, leaving gaps between the islets of cells connected with isthmuses and showed a reduced adhesion to plastic (Fig. 1c).

**Fig. 1.**
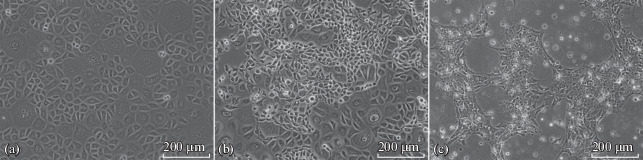
Phase contrast microscopy. Magnification, ×200. (a) Primary keratinocyte culture; (b) appearance of transformed keratinocytes after the immortalization procedure, CnT-07 medium; (c) transformed keratinocytes, DMEM/F12 medium with 10% serum.

As a result of a cytogenetic study, we detected a hyperdiploid set of chromosomes (modal number of chromosomes, 55–69) with multiple chromosomal abnormalities such as duplication of the p31–p32, p12–p36, and q25–q44 regions of the 1st chromosome, deletions of the q23-qter region of the long arm of the 3rd chromosome and the q31-qter region of the long arm of the 7th chromosome, additional material of unknown origin on the p-arm of the 4th chromosome and on the q-arm of the 13th chromosome, from 4 to 8 marker chromosomes. As in other immortalized keratinocyte lines, such as NM1 and NIKs, this cell line has chromosome 8 trisomy (tetrasomy in HaCaT), the increase in the number of genes in which is presumably responsible for the spontaneous immortalization of keratinocytes [5].

Immunofluorescent staining of the cell culture revealed the expression of the basal keratinocyte marker keratin 14 (K14) (Fig. 2a). Notably, the K14 expression pattern practically lacked the filament network. The cells also showed negative staining for keratin 5. It was previously reported that the expression of these keratins is a marker of oncotransformation and that both keratins are actively involved in maintaining the ability of cells to actively proliferate [9]. Therefore, the pathways promoting the transformation of the cells obtained by us have to be clarified in subsequent studies. The cells also showed negative staining for keratin 10 (K10) and transcription factor p63, and some of the cell nuclei are positively stained for telomerase (Fig. 2b).

**Fig. 2.**
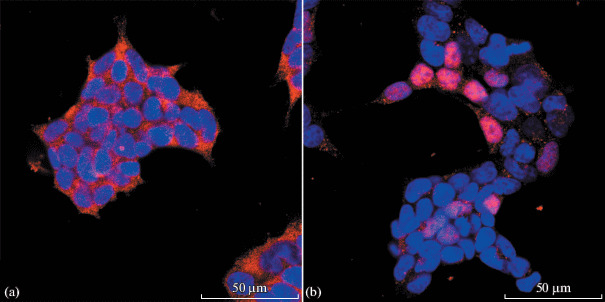
Transformed keratinocyte culture. Immunofluorescent staining. Confocal microscopy. Blue—DNA (DAPI); red—keratin 14 (a) or telomerase (b).

When transplanted into Nude Crl:NU(NCr)-Foxn1nu immunodeficient mice (Charles River, Germany), these cells formed tumors, in which telomerase-expressing cells are found (Fig. 3d). Among them, cells expressing K14 and K10, as well as loricrin, a marker of late stratification of the epidermis, are also detected (Figs. 3a, 3b, 3c). There are also large tissue formations without pronounced nuclear structures that are positive when stained with antibodies against human nuclear material. This may be a consequence of terminal differentiation of cells in the tumor. Similar stratification pattern was also noted by researchers earlier for the HaCaT spontaneously immortalized keratinocytes after their transplantation into immunodeficient mice [10].

**Fig. 3.**
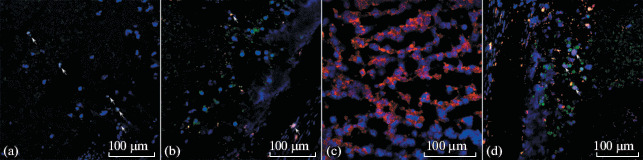
Section of the mouse testis after transplantation of transformed keratinocytes into it. Confocal microscopy. Blue—DNA (DAPI); green—human nuclei; red—keratin 14 (a), keratin 10 (b), loricrin (c), or telomerase (d). White arrows indicate the triple-stained cells.

During creation of skin equivalents, our immortalized keratinocytes behave in a similar way, regardless of the medium used. They form a sheet consisting of 15–30 cell layers. All cells in this sheet express K14, and cells in the upper layers and individual cells in the bulk express K10, similarly to HaCaT cells (Figs. 4b, 4e). At the same time, normal expression of collagen 7 is observed in the basal layer (Figs. 4c, 4f).

**Fig. 4.**
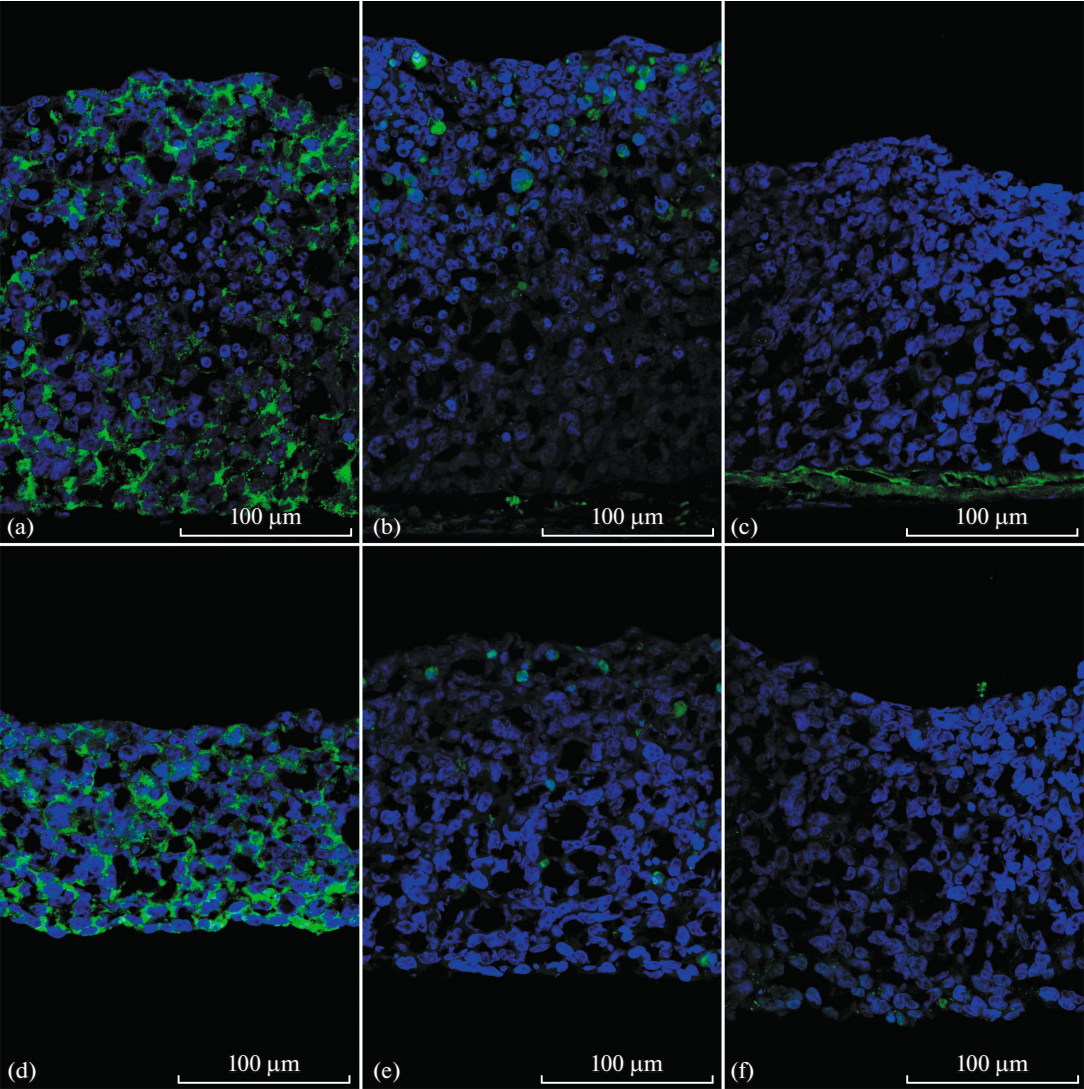
Sections of skin equivalents derived from transformed keratinocytes and primary human dermal fibroblasts. Confocal microscopy. (a, b, c) DMEM/F12 medium for stratification; (d, e, f) CnT-Prime Airlift medium. Blue—DNA (DAPI); green—keratin 14 (a, d), keratin 10 (b, e), or collagen 7 (c, f).

Using the TRAPEZE telomerase detection kit (Millipore), we showed that the level of telomerase activity in the new cell line derived by us is persists for almost 20 passages and is higher than in HaCaT and HeLa cells (Fig. 5). This, together with other changes, testifies to a profound transformation of cell metabolism.

**Fig. 5.**
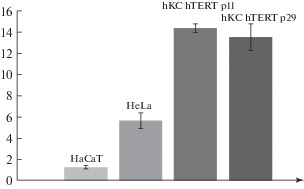
TRAPEZE test results. hKC hTERT—immortalized human keratinocytes. In the negative control (primary keratinocytes), telomerase activity is below the test sensitivity threshold. The ordinate shows telomerase activity (μmol/mg protein/min).

It can be assumed that, since the telomerase overexpression is not sufficient for keratinocyte immortalization, this cell line underwent genomic rearrangements, and additional epigenetic changes occurred. This allowed the cells of this culture, regardless of telomerase, to proceed to another state, which led to their immortalization.

This cell line can become a valuable tool in many studies related to investigation of the mechanisms of cell–cell interactions, tumorigenesis, and cell differentiation.
